# Lower Limits of Contact Resistance in Phosphorene Nanodevices with Edge Contacts

**DOI:** 10.3390/nano12040656

**Published:** 2022-02-16

**Authors:** Mirko Poljak, Mislav Matić, Tin Župančić, Ante Zeljko

**Affiliations:** Computational Nanoelectronics Group, Faculty of Electrical Engineering and Computing, University of Zagreb, HR 10000 Zagreb, Croatia; mislav.matic@fer.hr (M.M.); tin.zupancic@fer.hr (T.Ž.); ante.zeljko@fer.hr (A.Z.)

**Keywords:** phosphorene, black phosphorus, nanoribbon, edge contact, contact resistance, quantum transport, NEGF, metallization, broadening

## Abstract

Edge contacts are promising for improving carrier injection and contact resistance in devices based on two-dimensional (2D) materials, among which monolayer black phosphorus (BP), or phosphorene, is especially attractive for device applications. Cutting BP into phosphorene nanoribbons (PNRs) widens the design space for BP devices and enables high-density device integration. However, little is known about contact resistance (*R_C_*) in PNRs with edge contacts, although *R_C_* is the main performance limiter for 2D material devices. Atomistic quantum transport simulations are employed to explore the impact of attaching metal edge contacts (MECs) on the electronic and transport properties and contact resistance of PNRs. We demonstrate that PNR length downscaling increases *R_C_* to 192 Ω µm in 5.2 nm-long PNRs due to strong metallization effects, while width downscaling decreases the *R_C_* to 19 Ω µm in 0.5 nm-wide PNRs. These findings illustrate the limitations on PNR downscaling and reveal opportunities in the minimization of *R_C_* by device sizing. Moreover, we prove the existence of optimum metals for edge contacts in terms of minimum metallization effects that further decrease *R_C_* by ~30%, resulting in lower intrinsic quantum limits to *R_C_* of ~90 Ω µm in phosphorene and ~14 Ω µm in ultra-narrow PNRs.

## 1. Introduction

Two-dimensional (2D) materials are considered to be feasible candidates for future post-silicon electron devices due to their atomic thickness and exceptional mechanical, electronic, and carrier transport properties [1,2,3,4,5]. Among monoelemental 2D materials, monolayer black phosphorus (BP) or phosphorene is frequently identified as promising for future nanoscale field-effect transistors (FETs) due to its acceptable bandgap and carrier mobility that should enable appropriate switching and current-driving performance of phosphorene-based electron devices [6,7]. Recently, experimental demonstration and characterization results have been reported for micro-scale BP FETs [7,8,9], while theoretical and numerical simulation reports have been published for short-channel and wide-gate phosphorene FETs [4,10,11]. However, phosphorene nanoribbons (PNRs) that are quasi-one-dimensional phosphorene nanostructures are less explored, despite the opportunity provided by quantum confinement to adjust the material and device properties [12,13,14,15,16]. An additional motivation for further research on PNRs is provided by recent reports on fabricated and characterized ultra-narrow PNRs with the widths down to ~0.5 nm [17,18].

While 2D materials and their nanostructures seem promising for nanodevices, they suffer from high contact resistance (*R_C_*), which limits their performance and conceals their exceptional transport properties. For micro-scale BP FETs, *R_C_* was measured in the range from ≈1750 Ω µm [19] and ≈1100 Ω µm [9], over ≈700 Ω µm [20], down to ≈400 Ω µm [21] and 310 Ω µm [22]. Even the best reported *R_C_* values are unacceptably high for transistors in future high-density integrated circuits and, additionally, very little is known about *R_C_* levels and its behavior in PNR-based devices [23]. The most promising avenue toward low *R_C_* in 2D material-based devices seems to be the concept of edge contacts, i.e., one-dimensional contacts connected only at the edges of the nanostructure. Edge contacts are scalable, not limited by current transfer length as top contacts, and they allow the encapsulation of the 2D material that preserves its exceptional properties and enables long-term stability [24,25]. Almost all theoretical research on 2D material or nanoribbon-based FETs assumes ideal contacts, which only provides upper limits to device performance since the parasitic contact resistance is completely ignored [4,10,11].

In this work, we explore the contact resistance in PNRs with edge contacts using atomistic quantum transport simulations. We describe the metal electrodes by the wide-band limit model that is capable of reproducing metal-induced broadening and metallization effects. The impact of PNR size downscaling on *R_C_* is analyzed for technologically relevant PNR widths (<5.5 nm) and lengths (<16 nm). We reveal significant metallization effects visible in the deterioration of electronic and transport properties of PNRs, which are especially detrimental for nanoribbon lengths under ~8 nm. Contact resistance decreases with the width downscaling but increases considerably when the length decreases. Surprisingly, we show that even in the two-probe simulation setup there exists the optimum metal-nanoribbon interaction parameter that results in the minimum *R_C_* for a given PNR size. With metal edge contacts, optimum electrode material for PNRs is a more strongly-interacting metal in the case of longer devices, whereas ultra-short nanoribbons with lengths under ~6 nm demand contacts with a weaker interaction strength. Our results indicate that the quantum intrinsic limits of *R_C_*, i.e., minimum achievable *R_C_*, in large-area phosphorene devices could be as low as ~90 Ω µm. Moreover, an even lower *R_C_* of ~14 Ω µm can be obtained in 0.5 nm-wide PNRs with a careful choice of the electrode material. Our results give an encouraging perspective on the suitability of phosphorene and PNR FETs for future nanoscale electron devices and contribute towards theoretical understanding and practical minimization of contact resistance in nanodevices with edge contacts.

## 2. Methods

A multi-band tight-binding (TB) model from [26] is used for the construction of armchair PNR Hamiltonians that enter the retarded Green’s function within the non-equilibrium Green’s function (NEGF) formalism for quantum transport. This TB model agrees well with more advanced GW simulations for electron energies up to ~2 eV away from the Fermi level. While a more advanced Hamiltonian, e.g., one resulting from *ab initio* simulations, would improve the bandstructure accuracy in ultra-narrow PNRs [16], we choose a simpler model to reduce the computational burden since we investigate numerous devices of different sizes and contact-device interaction strengths. Regarding device size, we focus on technologically relevant extremely-scaled PNRs with the widths (*W*) under ~5.5 nm and lengths (*L*) below ~16 nm. The largest PNR under study consists of 2312 phosphorus atoms, and as many orbitals in the Hamiltonian matrix.

**Figure 1:**
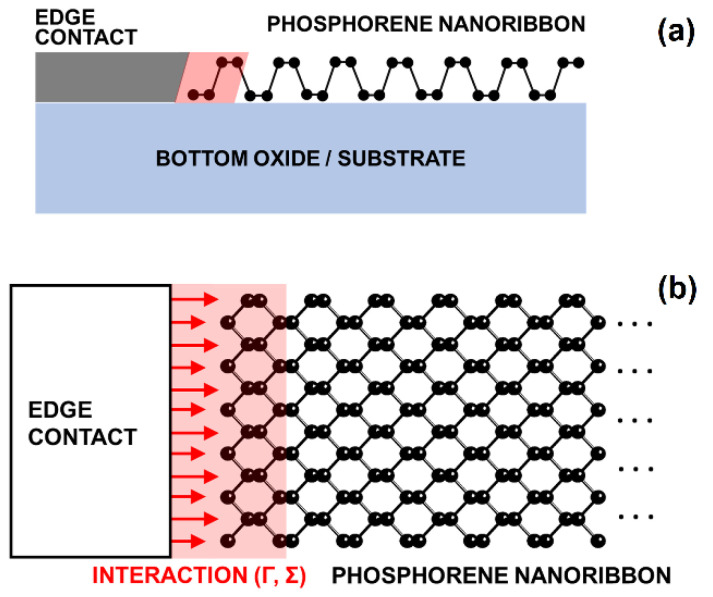
(**a**) Side-view and (**b**) top-view of the left metal edge contact attached to the PNR. Light red shaded area indicates the extent of metal-nanoribbon interaction across the closest super-cell, described by the broadening parameter (Γ) or contact-self energy (Σ).

Atomistic NEGF calculations are employed to investigate the electronic and transport properties of ultra-scaled PNRs, and to calculate contact resistance that emerges in PNRs after attaching metal edge contacts (MECs). Ballistic transport simulations are carried out by assuming a two-probe configuration, i.e., two MECs attached on the left and right edge of the nanoribbon, as illustrated in Figure 1. The NEGF formalism solves the Schrödinger’s equation for a given system with open boundary conditions (OBCs) [27,28]. The retarded Green’s function of the device is given by:

(1) $$G^{R}(E)={\left[\left(E+i0^{+}\right)I-H-\Sigma_{1}^{R}(E)-\Sigma_{2}^{R}(E)\right]}^{-1}$$

where *E* is the energy, *I* is the identity matrix, *H* is the device Hamiltonian, and Σ matrices are the retarded contact self-energies that account for OBCs in the nanoribbon imposed by the two attached MECs (left contact or contact 1, and right contact or contact 2). Our existing NEGF code, written in C/C++ and Compute Unified Device Architecture (CUDA) for heterogenous CPU-GPU execution, and previously demonstrated on graphene, silicene, germanene and phosphorene nanostructures [29,30], is used for calculations in this work.

Regarding the OBCs, we study the impact of attaching ideal and metal edge contacts on the electronic and transport properties of PNRs. The ideal edge contacts (IECs) are semi-infinite regions with the same geometry and bandstructure as the central region. Setting IECs in NEGF simulations is common throughout the literature, and this approach eliminates destructive interference at contact-device interfaces and results in perfect step-like transmission functions. In this work, MECs are treated with the wide-band limit (WBL) model in which only the constant imaginary part of contact self-energy matrices is retained [28]. As shown previously in the case of GNRs [31], we set the initial value of the nanoribbon-MEC interaction strength to −ImΣ*^R^* = 0.9 eV, in accordance with the average hopping parameter in the TB model for phosphorene, and with the expected density of states (DOS) in the model metal near the Fermi level [28,31]. We analyze the effects of changing −ImΣ*^R^* from the initial to lower and higher values, thus exploring the consequences of attaching weakly and strongly-interacting metals to the PNR, respectively. The MECs are assumed to be Ohmic so tunnel or Schottky barriers are disregarded to solely study the impact of metal-induced broadening or metallization effects. We note that our approach could be applied to large top contacts as well, but contact and device sizes are limited by the available computational facilities since atomistic NEGF simulations are very computationally intensive.

After transmission is calculated from the retarded Green’s function [32], we find the PNR conductance at 300 K from the expression:

(2) $$G=\frac{2e^{2}}{h}\int_{0}^{\infty }T(E)\left(-\partial f(E-E_{F})/\partial E\right)dE,$$

where *T*(*E*) is the transmission function, *f*(*E* − *E_F_*) is the Fermi–Dirac distribution function, *E_F_* is the Fermi level set to 50 meV away from the conduction band minimum, *e* is the electronic charge, and *h* is the Planck’s constant. Attaching WBL contacts induces broadening and decreases the transmission and conductance in the conduction and valence bands [31,33]. Therefore, by comparing the conductance values between the IEC and MEC cases, we can calculate the added contact resistance introduced by edge metal contacts using

(3) $$R_{C}=\frac{1}{G_{MEC}}-\frac{1}{G_{IEC}},$$

where *G_MEC_* and *G_IEC_* are PNR conductances with either metal or ideal edge contacts, respectively.

## 3. Results and Discussion

**Figure 2:**
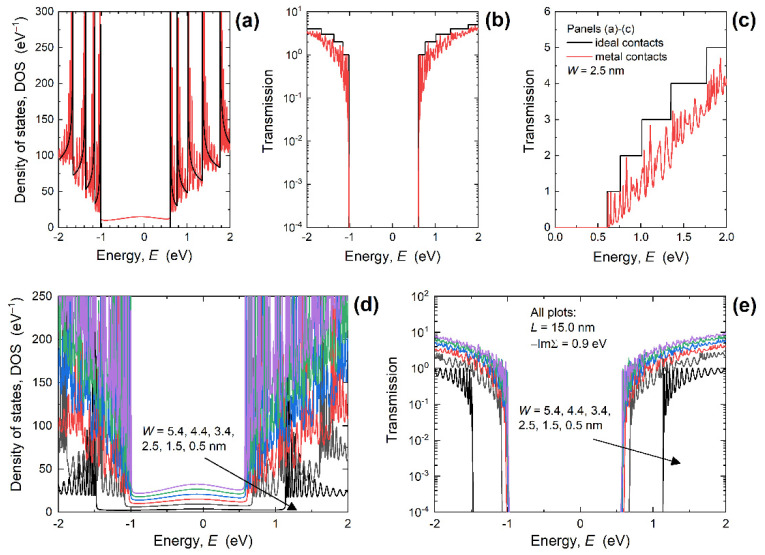
(**a**) DOS in linear, and transmission in (**b**) logarithmic and (**c**) linear scale for 15 nm-long and 2.5 nm-wide PNRs with ideal and metal edge contacts. Impact of width scaling on (**d**) DOS and (**e**) transmission in 15 nm-long PNRs with MECs. For all MEC-PNRs, −ImΣ*^R^* = 0.9 eV.

First, we focus on investigating the impact of PNR width downscaling from 5.4 nm to 0.5 nm on the electronic and transport properties of PNRs with IECs and MECs. Figure 2a shows the DOS in 2.5 nm-wide PNRs and we observe oscillations in DOS in the case of MECs, in contrast to van Hove singularities obtained for ideal contacts. These oscillations are known to occur in graphene and other nanodevices with metallic contacts [34], and can be easily understood from an analytical solution for a one-dimensional atomic chain. For the atomic chain, electron dispersion can be found to be *E*(*k*) = *E*_0_ + 2*t*cos(*ka*), where *k* is the wave-vector or crystal momentum, *E*_0_ is the local orbital energy, *t* is the hopping parameter, and *a* is the distance between atoms in the chain. Setting Σ_1_ = Σ_2_ = −iΓ/2 for the MEC case, where Γ is the broadening parameter, and using Equation (1) for the Green’s function, we analytically obtain the following spectral function:

(4) $$A(E)=G^{R}(\Gamma_{1}+\Gamma_{2})G^{A}=\frac{2\Gamma }{{\left(E-E_{0}\right)}^{2}+\Gamma^{2}}$$

where *G^A^* is the advanced Green’s function. Therefore, *A*(*E*) and density of states defined as DOS(*E*) = *A*(*E*)/π are clearly Lorentzian curves centered at *E*_0_, i.e., at band center, that decrease towards ban edges. This characteristic is in stark contrast to the case of ideal contacts that exhibits singularities at band edges and minimum DOS at the band center in 1D structures [27].

The impact of attaching MECs is also visible in the appearance of metal-induced gap states (MIGS) between the valence band maximum (VBM) and conduction band minimum (CBM). However, these states are clearly strongly localized as can be seen in Figure 2b that reports the transmission through the 2.5 nm-wide PNR with IECs and MECs. The transmission is extremely low inside the bandgap so the transport gap (*E_TG_*) exists. Therefore, MIGS do not contribute to transport, which is beneficial for FETs that need a transport gap to achieve efficient switching between the ON and OFF states. These findings demonstrate that PNRs are a more plausible solution of ultra-scaled FETs than GNRs given the considerable metallization-induced *E_TG_* decrease reported for GNRs with MECs [31]. While the energy gap of PNRs is immune to MEC-induced metallization effects, the characteristic shown in linear scale in Figure 2c demonstrates a significant transmission suppression by MECs. Lorentzian oscillations are also reported in the transmission as in the DOS curves, and the reasons are given in the Supplementary Materials—Supplementary Note S1. In contrast to ideal contacts that result in unitary transmission probability for each conducting mode and a step-like transmission function, attaching MECs described within the WBL model allows destructive interference for electron waves injected from the contact into the nanoribbon [31,33,34].

Figure 2d,e plots the DOS and transmission, respectively, for MEC PNRs with *L* = 15 nm and for various widths. When *W* decreases from 5.4 nm to 0.5 nm, the MIGS decrease in intensity due to shorter edge contacts in narrower nanoribbons. At the same time, reducing *W* increases the transport gap of PNRs, with Lorentzian oscillations existing in the transmission functions for all PNRs irrespective of the width. Therefore, in PNR nanodevices with MECs we expect a considerable deterioration of the current driving capabilities, even in the ballistic transport case that presents an upper intrinsic limit to device performance. Assessing device performance is beyond the scope of this work, but the presented data allows the calculation of relevant conductance values and enables the extraction of *R_C_* introduced by MECs, as described in Section 2.

**Figure 3:**
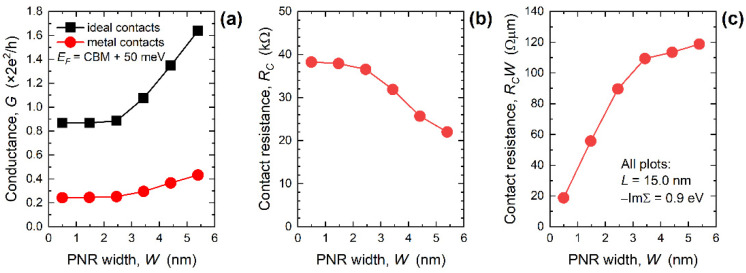
Width-dependence of (**a**) conductance, (**b**) contact resistance, and (**c**) width-normalized contact resistance in 15 nm-long PNRs. For all MEC-PNRs, −ImΣ*^R^* = 0.9 eV.

The influence of decreasing nanoribbon width on the conductance calculated for *E_F_* = CBM + 50 meV at 300 K is reported in Figure 3a for 15 nm-long PNRs. The conductance deteriorates in narrower devices due to lower transmission (see Figure 2e), which itself is a consequence of a lower number of modes or bands in narrower PNRs. In the case of IECs, the conductance decreases from 1.64 (constant 2*e*^2^/*h* is omitted for clarity) to 0.87 in the examined *W* range, whereas the conductance drops from 0.43 to 0.24 when MECs are connected to the PNRs. Comparing the two edge contact cases, we find that the conductance deterioration with MECs equals 74% for *W* = 5.4 nm and 72% in 0.5 nm-wide PNRs. Using Equation (3), we extract the contact resistance introduced by MECs and plot *R_C_* versus PNR width in Figure 3b. As the width decreases, *R_C_* increases from 22 kΩ (*W* = 5.4 nm) to 38.3 kΩ (*W* = 0.5 nm), which demonstrates that the narrower PNRs are more susceptible to MEC-induced metallization effects through the transmission deterioration. Figure 3c depicts the width-dependence of the width-normalized *R_C_*, i.e., *R_C_W*, which is a common contact resistance figure of merit for electron devices. In contrast to *R_C_* behavior in Figure 3b, *R_C_W* monotonically decreases with the downscaling of nanoribbon width, from 119 Ω µm for *W* = 5.4 nm down to 19 Ω µm for *W* = 0.5 nm.

In comparison to GNRs [31], phosphorene nanodevices exhibit a 62% higher *R_C_* for the widest nanoribbons, whereas the narrowest PNRs offer a 10% lower *R_C_* (at *W* ~ 0.5 nm). By extrapolating the results for wide nanoribbons to large-area 2D material devices, our results indicate that micro-scale phosphorene devices should have a significantly higher *R_C_* than graphene devices. This finding agrees with the literature that reports the best *R_C_* of 400 Ω µm [21] to 310 Ω µm [22] for phosphorene FETs, whereas the best reported *R_C_* for graphene FETs is ~80 Ω µm [35]. On the other hand, *R_C_* is very low in narrowest PNRs which means that patterning phosphorene into nanoribbons offers a promising avenue for *R_C_* minimization in ultra-scaled devices that enable high-density integration. The best reported experimental *R_C_* for phosphorene devices is ~300 Ω µm, so the space for improvement of the contact resistance exists and is quite extensive since the quantum limit of *R_C_W* reported above (~20 Ω µm) is more than 15× lower than the best-reported measured *R_C_W* value.

**Figure 4:**
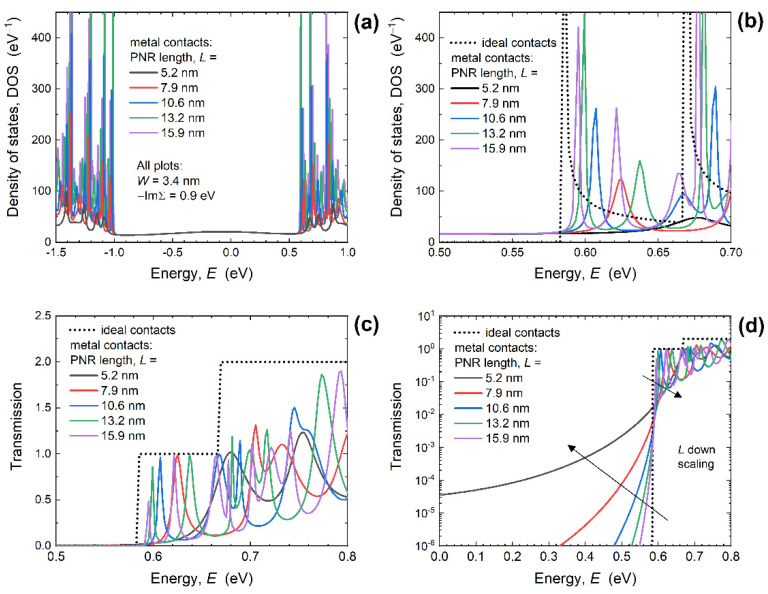
DOS in (**a**) entire energy range, and (**b**) in the conduction band for 3.4 nm-wide PNRs of various lengths with ideal and metal edge contacts. Influence of length downscaling on transmission in the (**c**) linear and (**d**) logarithmic scale. For all MEC-PNRs, −ImΣ*^R^* = 0.9 eV.

Width scaling provides an opportunity to expand the design space through confinement effects, however, length scaling is also important because in modern CMOS industry the channel length decrease is the main driving force behind FET performance improvement. Hence, in the following paragraphs we set a common *W* = 3.4 nm and analyze the electronic and transport properties, and *R_C_* for PNRs with the lengths from ~16 nm down to ~5 nm. Figure 4a shows the DOS of MEC-PNRs for various lengths with a zoomed-in energy range around the CBM reported in Figure 4b. The DOS again exhibits Lorentzians instead of van Hove singularities due to metallization effects. In the case of *L* scaling, MIGS are present but the magnitude of localized states inside the bandgap does not change with PNR length. In contrast, Figure 4b shows that DOS inside the conduction band changes considerably when *L* decreases, with the first DOS peak closest to CBM being shifted away from the CBM when the PNR length is scaled down. The CBM is positioned at *E* = 583 meV in the case of IECs, whereas the closest Lorentzian peak is situated at *E* = 595 meV for *L* = 15.9 nm in PNRs with MECs. Decreasing the length to 7.9 nm moves the first peak to *E* = 623 meV, while for *L* = 5.2 nm the first peak is positioned at *E* = 677 meV, i.e., shifted by 94 meV from the CBM. In addition to significant qualitative changes, we observe that DOS values decrease when *L* is scaled down, which is expected to decrease the ability of ultra-short PNRs to generate enough charge carriers for acceptable performance of PNR-based FETs.

As reported in Figure 4c, the transmission curves exhibit variations similar to those seen in the DOS. Decreasing *L* reduces the number of Lorentzians and shifts the first peak away from the CBM. The transmission is greatly reduced in PNRs with MECs, which is especially evident for *L* = 5.2 nm for which the transmission is almost completely suppressed in the entire energy range corresponding to the first transmission step of the PNR with IECs. Results presented in Figure 4c seem to indicate that the downscaling of the length of PNRs with MECs leads to the increase of the transmission gap, but Figure 4d that reports the transmission in logarithmic scale reveals a more complicated picture. Namely, while the transmission decreases with *L* downscaling in the energy range above the CBM, the opposite is true inside the bandgap. As the PNR length decreases, transmission probability below the CBM increases considerably, which leads to the contraction of the transport gap. If we define the transport gap as the energy range where the transmission is lower than 0.001, we find that *E_TG_* decreases by 56 meV when *L* = 7.9 nm and by 252 meV in the 5.2 nm-long PNR. Since the existence and value of *E_TG_* is very important for the practical realization of FETs, this finding clearly shows that broadening or metallization effects must be included into the physical framework used for the simulation of nanoscale electron devices. We have previously reported that in ultra-short GNRs the transport gap closes completely due to these metallization effects [31], but PNRs are evidently more resilient to the influence of metal edge contacts than GNRs since *E_TG_* still exists, albeit being somewhat smaller.

**Figure 5:**
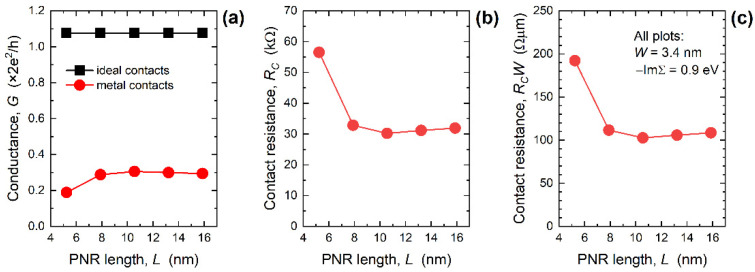
Length-dependence of (**a**) conductance, (**b**) contact resistance, and (**c**) width-normalized contact resistance in 3.4 nm-wide PNRs. For all MEC-PNRs, −ImΣ*^R^* = 0.9 eV.

The observed strong suppression of transmission near the CBM consequently decreases the conductance and induces contact resistance at the two MEC-nanoribbon interfaces. Figure 5a reports the conductance calculated for *E_F_* = CBM + 50 meV and 300 K versus PNR length for 3.4 nm-wide PNRs with IECs and MECs. While the conductance is length-independent in the case of ideal contacts, it noticeably decreases for *L* < 8 nm when MECs are attached to PNRs. Hence, conductance difference between the two contact configurations is largest in the shortest devices, which is also seen in the extracted *R_C_* shown in Figure 5b. As the length is downscaled, *R_C_* increases from 31.9 kΩ (*L* = 15.9 nm) to 56.6 kΩ (*L* = 5.2 nm), which is a consequence of the greatly decreased transmission near the CBM in 5.2 nm-long PNRs (see Figure 4c). After width-normalization the contact resistance curve in Figure 5c stays qualitatively the same as in Figure 5b. The *R_C_W* equals 109 Ω µm for *L* = 15.9 nm, stays almost constant down to *L* = 7.9 nm, and then increases to 192 Ω µm in MEC-PNRs that are only 5.2 nm long. In addition to transport gap decrease, the observed boost of *R_C_W* is yet another negative consequence of attaching metal contacts if we consider ultra-scaled PNRs as channel material for future FETs.

All the results considered so far are based on using −ImΣ*^R^* = 0.9 eV in MEC self-energy matrices, which presents a model metal material with moderately-strong interactions with the nanoribbon. However, we have recently shown for FETs based on various monoelemental 2D materials that there exists the optimum interaction parameter value leading to the lowest transmission decrease, which enables the minimization of *R_C_* in such nanodevices [23]. An example concerning optimum −ImΣ*^R^* for transmission is given for a 2.45 nm-wide and 15 nm-long PNR in the Supplementary Materials—Supplementary Note S2, Figure S1. This finding is in accordance with the study dealing with reflections and transmissions in atomic chains and carbon nanotubes connected to wide-band leads reported in [36]. An illustrative example based on 1D atomic chains about the evolution of eigenstates and transmission functions, and the existence of the optimum interaction parameter is provided in Supplementary Materials—Supplementary Note S3, Figures S2–S4. For phosphorene FETs with 15 nm-long channels, the optimum −ImΣ*^R^* of ~2 eV was reported in [23]. Therefore, it seems reasonable to assume that such optimum interaction parameters will exist also in the case of a two-probe setup assessed in this work with the aim of finding quantum limits of *R_C_* in PNRs with metal edge contacts. In the following discussions, we calculate *R_C_W* for MEC-PNRs of various dimensions, and for −ImΣ*^R^* that ranges from 0.01 eV to 20 eV. The −ImΣ*^R^* value range is chosen according to studies on graphene-metal and carbon nanotube-metal contacts in [37,38]. While we do not perform *ab initio* interface studies for phosphorene-metal systems as in [39,40,41] due to heavy computational burden of doing so for a large variety of nanoribbon sizes and metal choice, the WBL approach allows us to explore the impact of weakly, moderately and strongly-interacting metal electrodes on the contact resistance in PNR nanodevices. Generally, the low −ImΣ*^R^* values in our approach correspond to weakly interacting metals such as Al, Ag, Au, Cu, and higher −ImΣ*^R^* values describe strongly interacting metals such as Cr, Ni, Pd, Ti, where the interaction strength is assessed in detail by *ab initio* calculations in [39].

**Figure 6:**
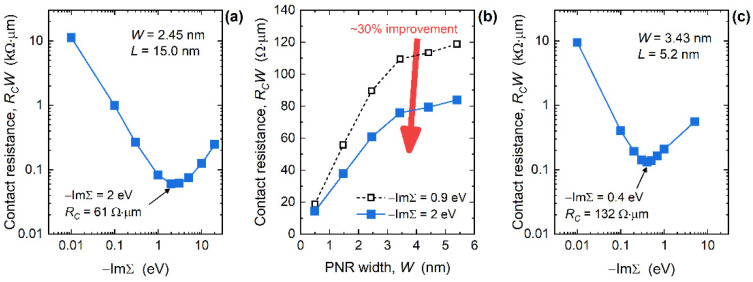
(**a**) Dependence of width-normalized contact resistance on interaction strength in 2.45 nm-wide and 15 nm-long PNRs with MECs. (**b**) Impact of PNR width downscaling on *R_C_W* for the two −ImΣ*^R^* values, initial and the optimum one. (**c**) *R_C_W* versus interaction strength in 3.43 nm-wide and 5.2 nm-long PNRs with MECs.

Figure 6a reports the dependence of *R_C_W* on the interaction strength in 2.45 nm-wide and 15 nm-long phosphorene nanoribbons. Starting from very weakly interacting metals (−ImΣ*^R^* = 0.01 eV) where *R_C_W* = 11.2 kΩ µm, the resistance first decreases and reaches a minimum of 61 Ω µm for the optimum −ImΣ*^R^* of 2 eV, and then increases to 247 Ω µm when strongly-interacting (−ImΣ*^R^* = 20 eV) MECs are attached to the PNR. In comparison to the initial resistance value of 90 Ω µm for *W* = 2.45 nm in Figure 3c, a careful choice of contact material can reduce *R_C_W* by 32%. Assuming the optimum interaction parameter of 2 eV for all 15 nm-long devices, in Figure 6b, we report *R_C_W* values for the entire examined PNR width range. In this case the resistance decreases from 84 Ω µm for W = 5.4 nm down to 14 Ω µm in the 0.5 nm-wide MEC-PNR. The improvement is almost constant and equals ~30% for nanoribbon widths down to ~1.5 nm, whereas *R_C_W* drops by 23% for 0.5 nm-wide PNRs with MECs and −ImΣ*^R^* = 2 eV. The characteristics reported in Figure 6b indicate that a minimum *R_C_W* of ~90 Ω µm is achievable in large-area phosphorene devices with edge contacts, which puts these lower quantum limits of *R_C_* in phosphorene close to the best reported contact resistance in graphene devices [24,35]. In addition, our results show that the contact resistance can be further minimized to ~14 Ω µm by using ultra-narrow PNRs as channel material in ultra-scaled FETs.

Concerning the PNR length scaling, optimum −ImΣ*^R^* stays the same down to about *L* = 10 nm (not shown), and then decreases. Figure 6c plots *R_C_W* versus the interaction parameter −ImΣ*^R^* for 3.4 nm-wide PNRs with a length of 5.2 nm. For this device, *R_C_W* starts at 5.5 kΩ µm in the case of weakly-interacting WBL edge contacts, then decreases and reaches the optimum value of 132 Ω µm for −ImΣ*^R^* = 0.4 eV, after which *R_C_W* increases to 559 Ω µm for the strongest-interacting MECs considered. The improvement of *R_C_W* in 5.2 nm-long PNRs with −ImΣ*^R^* = 0.4 eV, over the initial case where −ImΣ*^R^* was 0.9 eV, amounts to 31%. Hence, even in the shortest devices, the contact resistance can be significantly reduced despite the very strong metallization-induced effects. Nevertheless, the choice of optimum electrode material changes in shorter devices that clearly benefit from less-interacting MECs (optimum −ImΣ*^R^* = 0.4 eV) than longer PNRs (optimum −ImΣ*^R^* = 2 eV) and, by extrapolation, large-area phosphorene devices with edge contacts.

## 4. Conclusions

Using atomistic quantum transport simulations, we studied the consequences of attaching metal electrodes in the edge-contact configuration on the electronic and transport properties of ultra-scaled PNRs. Since we ignore tunnel and Schottky barriers, our approach allows us to explore upper performance limits and lower limits on contact resistance in these devices. Attaching MECs leads to Lorentzian peaks in the DOS and transmission characteristics, appearance of localized MIGS inside the bandgap, noticeable narrowing of the transport gap, and overall suppression of the transmission in the conduction and valence bands. This suppression decreases the device conductance and introduces additional contact resistance at electrode-nanoribbon interfaces. We have shown that PNR width downscaling in the 5.4–0.5 nm range decreases *R_C_W* from 119 Ω µm down to 19 Ω µm. Therefore, patterning phosphorene into PNRs provides a compelling way to minimize contact resistance to levels acceptable to the CMOS industry for nanoscale FETs. In contrast to width scaling, *R_C_W* increases with decreasing PNR length from 109 Ω µm when *L* = 15.9 nm to 192 Ω µm in 5.2 nm-long PNRs with MECs. In addition to *E_TG_* decrease, the boosted *R_C_W* in ultra-short PNRs also limits their feasibility as channel material in ultra-scaled FETs, and emphasizes the importance of including metallization effects in device simulation at this scale. Finally, we have demonstrated the existence of optimum interaction parameters or optimum electrode materials that can significantly improve *R_C_W* (30% in comparison to the initial case of −ImΣ*^R^* = 0.9 eV). Surprisingly, shorter PNRs favor less-interacting metals (optimum −ImΣ*^R^* = 0.4 eV), whereas longer PNRs profit from more strongly interacting electrodes (optimum −ImΣ*^R^* = 2 eV) that reduce *R_C_W* to very low levels, i.e., ~14 Ω µm in the narrowest PNRs. Our work proves that there is enough room for *R_C_W* improvement in BP and PNR devices since quantum limits of *R_C_W* reported in this work are an order of magnitude lower than the best reported measured contact resistance. Regarding large-area phosphorene devices with edge contacts, we show that *R_C_W* of ~90 Ω µm is achievable, which is close to the best reported contact resistance in graphene devices.

## Data Availability

The data presented in this study are contained within the article and are available on request from the corresponding author.

## Supplementary Materials

The following supporting information can be downloaded at: https://www.mdpi.com/article/10.3390/nano12040656/s1, pdf document containing additional simulation results, mainly on 1D atomic chains to illustrate the emergence of contact resistance and existence of optimum electrode material in nanodevices with edge contacts: Figure S1: Transmission of 15 nm-long and 2.45 nm-wide PNRs with MECs for various contact self-energy or interaction parameter values ranging from 0.09 eV to 20 eV, Figure S2: Transmission function of a 5-atom chain with ideal and metal contacts for different ratios of the contact self-energy and inter-atomic hopping parameter −Im{Σ}/*t* that ranges from 0.1 to 10, Figure S3: The same as in Figure S2 but for −Im{Σ}/*t* values that range from 0.9 to 1.5, Figure S4: Transmission in a 5-atom chain for weakly and very strongly interacting MECs, and dependence of eigenvalues on the interaction strength.
