# Supplementary material for: Lower Limits of Contact Resistance in Phosphorene Nanodevices with Edge Contacts
Source: Nanomaterials (Basel). 2022 Feb 16;12(4):656. doi: 10.3390/nano12040656 (PMC8874988; doi:10.3390/nano12040656)
Supplement: Supplementary file 1 [file nanomaterials-12-00656-s001.zip › nanomaterials-1592448-supplementary.pdf]

nanomaterials

Supplementary Materials

Lower Limits of Contact Resistance in Phosphorene Nanodevices with Edge Contacts

Mirko Poljak *, Mislav Matić, Tin Župančić and Ante Zeljko

Computational Nanoelectronics Group, Faculty of Electrical Engineering and Computing, University of Zagreb, HR 10000 Zagreb, Croatia; mislav.matic@fer.hr (M.M.); tin.zupancic@fer.hr (T.Ž.); ante.zeljko@fer.hr (A.Z.)
* Correspondence: mirko.poljak@fer.hr

Supplementary Note S1

The Shape of the Transmission Function in Atomic Chains with Metal Edge Contacts

In this Note we analytically find the shape of the transmission function in a 1D atomic-chain structure connected to WBL metal edge contacts (MECs), i.e., we explain the origin of Lorentzians in the transmission reported in the main text.

In the ideal edge contact (IEC) case, we can assume that the "channel" consists of a single atomic site with a single orbital and zero on-site orbital energy, while the contacts are semi-infinite 1D atomic chains. Interaction (hopping) parameter is designated by t. In the IEC case, the dispersion and contact self-energy are

$$E(k) = E_0 - 2t\cos(ka) \quad \text{and} \quad \Sigma = -t\exp(+ika)$$

where a is inter-atomic distance and E_0 = 0 eV is the on-site energy or band-center for the dispersion relation. Band-width is equal to $4t$, i.e., the band exists within $-2t \le E \le 2t$. The Green's function of the "channel" then equals

$$\begin{aligned} G(E) &= \left[E - H - \Sigma_L - \Sigma_R\right]^{-1} = \\ &= \left[E - 0 + 2t\exp(+ika)\right]^{-1} = \\ &= \left[-2t\cos(ka) - 0 + 2t\cos(ka) + \mathrm{i}2t\sin(ka)\right]^{-1} = \frac{1}{\mathrm{i}2t\sin(ka)} \end{aligned}$$

while for broadening we obtain

$$\Gamma_{L,R} = -2\,\mathrm{Im}\,\Sigma_{L,R} = 2t\sin(ka)$$

Therefore, the well-known unitary transmission is obtained in the case of ideal contacts

$$\bar{T}(E) = trace\left(\mathbf{\Gamma}_L\mathbf{G}\mathbf{\Gamma}_R\mathbf{G}^\dagger\right) = 2t\sin(ka)\frac{1}{\mathrm{i}2t\sin(ka)}2t\sin(ka)\frac{-1}{\mathrm{i}2t\sin(ka)} = 1$$

for the entire band width, i.e., for $-2t \le E \le 2t$.

In the case of MECs, broadening is constant, i.e.

$$\Sigma_L = \Sigma_R = -\mathrm{i}\,\Gamma/2$$

so the Green's function of the "channel" becomes

$$\begin{aligned} G(E) &= \left[E - H - \Sigma_L - \Sigma_R\right]^{-1} = \\ &= \left[E + \mathrm{i}\Gamma\right]^{-1} \end{aligned}$$

With MECs connected to the "channel", its transmission function becomes a Lorentzian

$$\bar{T}(E) = trace\left(\mathbf{\Gamma}_L \mathbf{G} \mathbf{\Gamma}_R \mathbf{G}^\dagger\right) = \Gamma \frac{1}{E + \mathrm{i}\Gamma} \Gamma \frac{1}{E - \mathrm{i}\Gamma} = \frac{1}{1 + \left(\dfrac{E}{\Gamma}\right)^2}$$

with a maximum of one but only at band center (i.e., at $E = E_0 = 0$). Going away from the band-center, the transmission decreases and for $E = \Gamma$ the transmission equals 0.5. Moreover, transmission is non-zero beyond the ideal band-width $-2t \le E \le 2t$, because at band edges, $E = \pm 2t$, the transmission equals 0.2 when $\Gamma = t$.

When Γ decreases (weaker interaction between the channel and MECs), transmission curve becomes narrower which decreases transmission probability in the band. However, when Γ increases (stronger interaction between the channel and MECs), transmission curve broadens thus increasing transmission probability over a wider energy range. Nevertheless, in cases other than 1D atomic chains that are easily solved analytically, such as 2D material nanoribbons, this broadening opens the possibility of interaction with other conducting modes, which can lead to destructive or constructive interference of the injected electron waves and, consequently, it can lead to a reduced or increased transmission for certain energies.

Supplementary Note S2

The Existence of the Optimum Interaction Parameter for Phosphorene Nanoribbons with Metal Edge Contacts

Figure S1 reports the transmission functions in the conductance band for a PNR with MECs attached, for several values of the interaction parameter $-\mathrm{Im}\{\Sigma\}$. For moderate self-energy values (i.e., ≈1–3 eV) the transmission curves are very similar. In contrast, transmission probability deteriorates considerably as $-\mathrm{Im}\{\Sigma\}$ either increases or decreases. These findings indicate that there exists the optimum contact material, represented by the optimum interaction parameter, which consequently results in the minimum contact resistance.

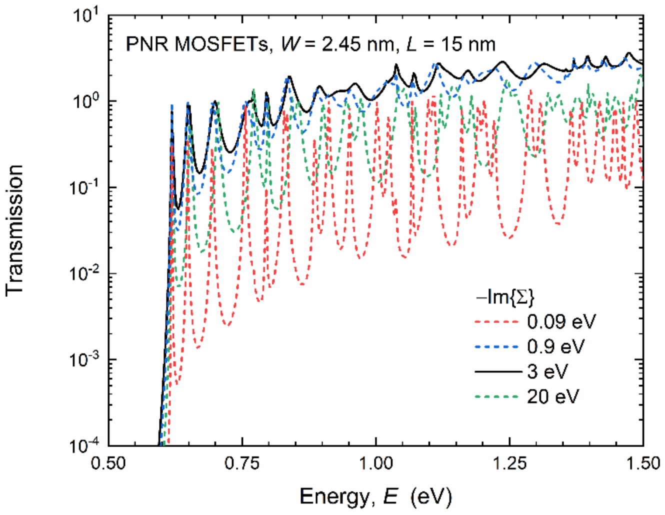

Figure S1. Transmission of 15 nm-long and 2.45 nm-wide PNRs with MECs for various contact self-energy or interaction parameter values ranging from 0.09 eV to 20 eV.

Supplementary Note S3

Evolution of the Transmission and Eigenstates with Increasing Interaction Strength in Atomic Chains with Metal Edge Contacts

Figure S2 shows the transmission functions of the 5-atom chain for various $-\mathrm{Im}\{\Sigma\}/t$ values. Maximum transmission over the widest possible energy range in the vicinity of the band-center (E = 0 eV) is obtained in Figure S2d for the case $-\mathrm{Im}\{\Sigma\}/t = 1$. A closer look is provided by Figure S3 that shows a similar plot, but for $-\mathrm{Im}\{\Sigma\}/t$ values around the previously found optimum of 1, ranging from 0.9 to 1.5. This closer look reveals that $-\mathrm{Im}\{\Sigma\}/t$ needed for optimum transmission is closer to 1.1, since for lower ratios the transmission becomes oscillatory, and for larger ratios the unitary transmission band-width becomes narrower. Therefore, optimum interaction parameter is determined by the bandstructure of the material to which the MECs are attached.

Figure S4 plots the evolution of the transmission function and eigenstates in the 5-atom chain with the increase of the interaction parameter. In the case of weakly-interacting MECs, i.e., $-\mathrm{Im}\{\Sigma\}/t = 0.1$, the transmission exhibits five peaks that correspond to five eigenstates of the 5-atom chain. As shown in Figure S4b, the number of eigenstates change when the interaction strength increases from five (weakly interacting MECs) to only three (strongly interacting MECs). Therefore, for strongly interacting metal contacts, i.e., $-\mathrm{Im}\{\Sigma\}/t = 10$, the transmission exhibits three peaks at the three new eigenstates shifted by the MEC-induced broadening.

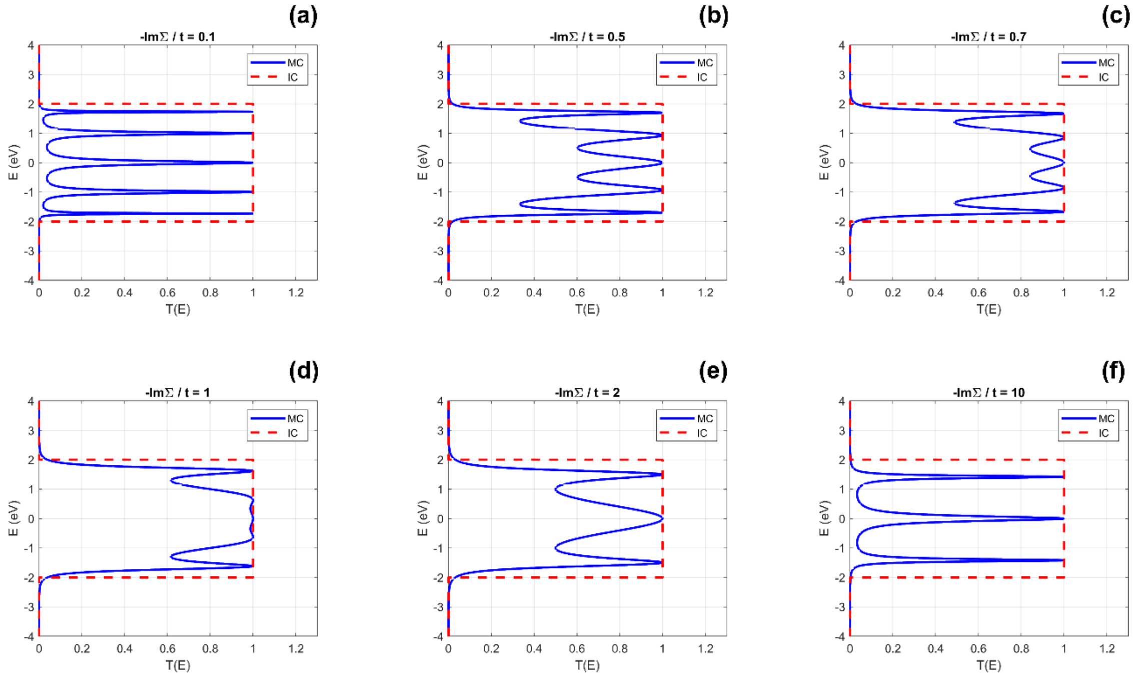

Figure S2. Transmission function of a 5-atom chain with ideal and metal contacts for different ratios of the contact self-energy and inter-atomic hopping parameter $-\mathrm{Im}\{\Sigma\}/t$ that equals (**a**) 0.1, (**b**) 0.5, (**c**) 0.7, (**d**) 1, (**e**) 2, and (**f**) 10.

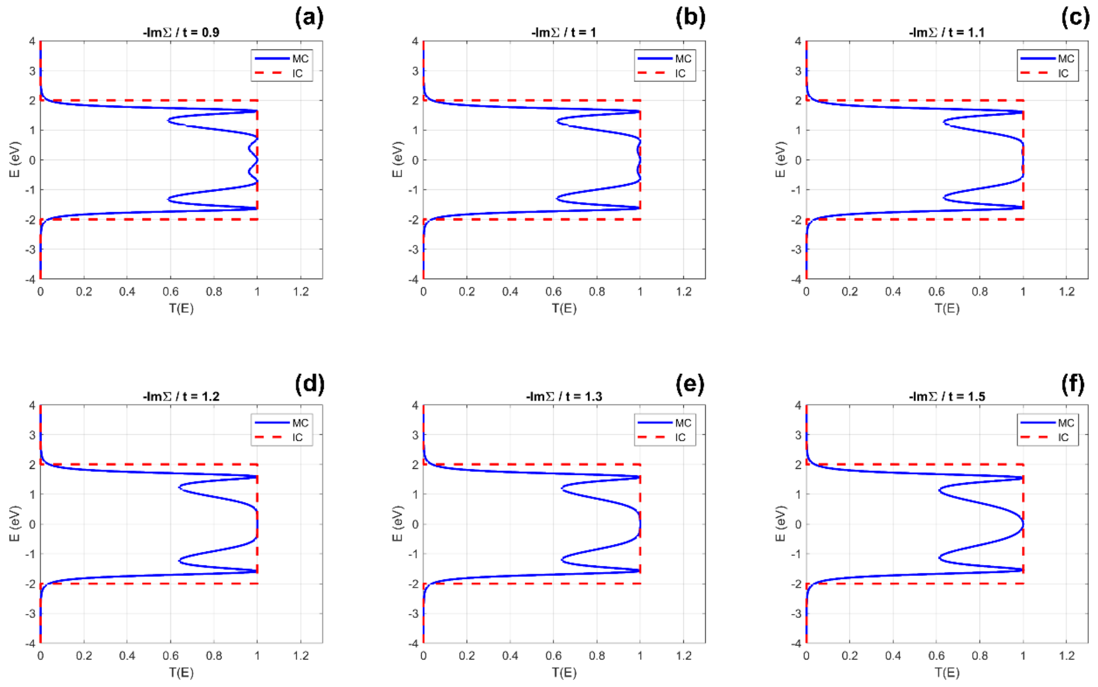

Figure S3. The same as in Figure S2 but for $-\mathrm{Im}\{\Sigma\}/t$ values that equal (**a**) 0.9, (**b**) 1, (**c**) 1.1, (**d**) 1.2, (**e**) 1.3, and (**f**) 1.5.

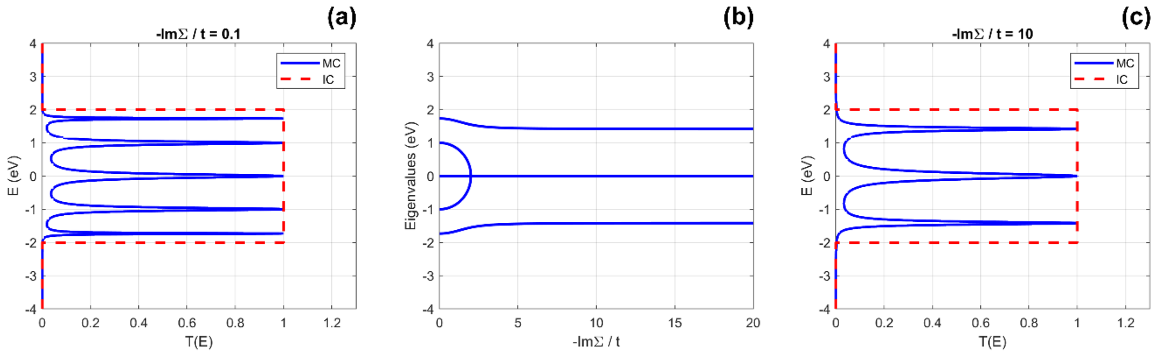

Figure S4. Transmission in a 5-atom chain for (**a**) weakly interacting, and (**c**) very strongly interacting MECs. (**b**) Dependence of eigenvalues on the interaction strength.
